# An extension of a multidimensional Hilbert-type inequality

**DOI:** 10.1186/s13660-017-1355-6

**Published:** 2017-04-18

**Authors:** Jianhua Zhong, Bicheng Yang

**Affiliations:** Department of Mathematics, Guangdong University of Education, Guangzhou, Guangdong 510303 P.R. China

**Keywords:** 26D15, 47A05, Hilbert-type inequality, weight coefficient, equivalent form, operator, norm

## Abstract

In this paper, by the use of weight coefficients, the transfer formula and the technique of real analysis, a new multidimensional Hilbert-type inequality with multi-parameters and a best possible constant factor is given, which is an extension of some published results. Moreover, the equivalent forms, the operator expressions and a few particular inequalities are considered.

## Introduction

If $p > 1$, $\frac{1}{p} + \frac{1}{q} = 1$, $a_{m},b_{n} \ge0$, $a = \{ a_{m}\}_{m = 1}^{\infty} \in l^{p}$, $b = \{ b_{n}\}_{n = 1}^{\infty} \in l^{q}$, $\Vert a \Vert _{p} = (\sum_{m = 1}^{\infty} a_{m}^{p} )^{\frac{1}{p}} > 0$, $\Vert b \Vert _{q} > 0$, then we have the following Hardy-Hilbert inequality with the best possible constant $\frac{\pi}{\sin(\pi/p)}$:

1 $$ \sum_{m = 1}^{\infty} \sum _{n = 1}^{\infty} \frac{a_{m}b{}_{n}}{m + n} < \frac{\pi}{\sin(\pi/p)} \Vert a \Vert _{p} \Vert b \Vert _{q}, $$

and the following Hilbert-type inequality:

2 $$ \sum_{m = 1}^{\infty} \sum _{n = 1}^{\infty} \frac{a_{m}b{}_{n}}{\max\{ m,n\}} < pq \Vert a \Vert _{p} \Vert b \Vert _{q} $$

with the best possible constant factor *pq* (cf. [1], Theorem 315, Theorem 341). Inequalities (1) and (2) are important in the analysis and its applications (cf. [1–3]).

Assuming that $\{ \mu_{m}\}_{m = 1}^{\infty}$, $\{ \nu_{n}\}_{n = 1}^{\infty}$ are positive sequences,

$$U_{m} = \sum_{i = 1}^{m} \mu_{i},\quad\quad V_{n} = \sum _{j = 1}^{n} \nu_{j}\quad \bigl(m,n \in \mathbb{N} = \{ 1,2,\ldots\} \bigr), $$

we have the following Hardy-Hilbert-type inequality (cf. [1], Theorem 321):

3 $$ \sum_{m = 1}^{\infty} \sum _{n = 1}^{\infty} \frac{a_{m}b{}_{n}}{U_{m} + V_{n}} < \frac{\pi}{\sin(\pi/p)} \Biggl( \sum_{m = 1}^{\infty} \frac{a_{m}^{p}}{m^{p - 1}} \Biggr)^{\frac{1}{p}} \Biggl( \sum_{n = 1}^{\infty} \frac{b_{n}^{q}}{n^{q - 1}} \Biggr)^{\frac{1}{q}}. $$

For $\mu_{i} = \nu_{j} = 1$ ($i,j \in\mathbb{N}$), inequality (3) reduces to (1).

In 2014, Yang and Chen [4] gave the following multidimensional Hilbert-type inequality: For $i_{0},j_{0} \in\mathbb{N}$, $\alpha,\beta> 0$,

$$\begin{aligned}& \Vert x \Vert _{\alpha}: = \Biggl( \sum_{k = 1}^{i_{0}} \bigl\vert x^{(k)} \bigr\vert ^{\alpha} \Biggr)^{\frac{1}{\alpha}} \quad \bigl(x = \bigl(x^{(1)},\ldots,x^{(i_{0})} \bigr) \in \mathbb{R}^{i_{0}} \bigr), \\& \Vert y \Vert _{\beta}: = \Biggl( \sum_{k = 1}^{j_{0}} \bigl\vert y^{(k)} \bigr\vert ^{\beta} \Biggr)^{\frac{1}{\beta}} \quad \bigl(y = \bigl(y^{(1)},\ldots,y^{(j_{0})} \bigr) \in \mathbb{R}^{j_{0}} \bigr), \end{aligned}$$

$0 < \lambda_{1} + \eta\le i_{0}$, $0 < \lambda_{2} + \eta\le j_{0}$, $\lambda_{1} + \lambda_{2} = \lambda$, $a_{m},b_{n} \ge0$, we have

4 $$ \begin{aligned}[b] &\sum_{n} \sum _{m} \frac{(\min\{ \Vert m \Vert _{\alpha}, \Vert n \Vert _{\beta} \} )^{\eta}}{(\max\{ \Vert m \Vert _{\alpha }, \Vert n \Vert _{\beta} \} )^{\lambda+ \eta}} a_{m}b_{n} \\ &\quad < K_{1}^{\frac{1}{p}}K_{2}^{\frac{1}{q}} \biggl[ \sum_{m} \Vert m \Vert _{\alpha}^{p(i_{0} - \lambda_{1}) - i_{0}}a_{m}^{p} \biggr]^{\frac{1}{p}} \biggl[ \sum_{n} \Vert n \Vert _{\beta}^{q(j_{0} - \lambda_{2}) - j_{0}}b_{n}^{q} \biggr]^{\frac{1}{q}}, \end{aligned} $$

where$\sum_{m} = \sum_{m_{i_{0}} = 1}^{\infty}\cdots\sum_{m_{1} = 1}^{\infty}$, $\sum_{n} = \sum_{n_{j_{0}} = 1}^{\infty}\cdots\sum_{n_{1} = 1}^{\infty}$, the series on the right-hand side are positive, and the best possible constant factor $K_{1}^{\frac{1}{p}}K_{2}^{\frac{1}{q}}$ is indicated by

$$K_{1}^{\frac{1}{p}}K_{2}^{\frac{1}{q}} = \biggl[ \frac{\Gamma^{j_{0}} ( \frac{1}{\beta} )}{\beta^{j_{0} - 1}\Gamma ( \frac{j_{0}}{\beta} )} \biggr]^{\frac{1}{p}} \biggl[ \frac{\Gamma^{i_{0}} ( \frac{1}{\alpha} )}{\alpha^{i_{0} - 1}\Gamma ( \frac{i_{0}}{\alpha} )} \biggr]^{\frac{1}{q}}\frac{\lambda+ 2\eta}{ (\lambda_{1} + \eta)(\lambda_{2} + \eta)}. $$

For $i_{0} = j_{0} = \lambda= 1$, $\eta= 0$, $\lambda_{1} = \frac{1}{q}$, $\lambda_{2} = \frac{1}{p}$, inequality (4) reduces to (2). The other results on this type of inequalities were provided by [5–17].

In 2015, Shi and Yang [18] gave another extension of (2) as follows:

5 $$ \sum_{m = 1}^{\infty} \sum _{n = 1}^{\infty} \frac{a_{m}b{}_{n}}{\max\{ U_{m},V_{n}\}} < pq \Biggl( \sum _{m = 1}^{\infty} \frac{a_{m}^{p}}{m^{p - 1}} \Biggr)^{\frac{1}{p}} \Biggl( \sum_{n = 1}^{\infty} \frac{b_{n}^{q}}{n^{q - 1}} \Biggr)^{\frac{1}{q}}. $$

Some other results on Hardy-Hilbert-type inequalities were given by [19–25].

In this paper, by the use of weight coefficients, the transfer formula and the technique of real analysis, a new multidimensional Hilbert-type inequality with multi-parameters and a best possible constant factor is given, which is an extension of (4) and (5). Moreover, the equivalent forms, the operator expressions and a few particular inequalities are considered.

## Some lemmas

If $\mu_{i}^{(k)} > 0$ ($k = 1,\ldots,i_{0}$; $i = 1,\ldots,m$), $\nu _{j}^{(l)} > 0$ ($l = 1,\ldots,j_{0}$; $j = 1,\ldots,n$), then we set

6 $$\begin{aligned}& U_{m}^{(k)}: = \sum_{i = 1}^{m} \mu_{i}^{(k)}\quad (k = 1,\ldots,i_{0}),\quad \quad V_{n}^{(l)}: = \sum_{j = 1}^{n} \nu_{j}^{(l)}\quad (l = 1,\ldots,j_{0}), \\& U_{m} = \bigl(U_{m}^{(1)}, \ldots,U_{m}^{(i_{0})} \bigr),\quad\quad V_{n} = \bigl(V_{n}^{(1)}, \ldots,V_{n}^{(j_{0})} \bigr) \quad (m,n \in\mathbb{N}). \end{aligned}$$

We also set functions $\mu_{k}(t): = \mu_{m}^{(k)}$, $t \in(m - 1,m]$ ($m \in \mathbb{N}$); $\nu_{l}(t): = \nu_{n}^{(l)}$, $t \in(n - 1,n]$ ($n \in \mathbb{N}$), and

7 $$\begin{aligned}& U_{k}(x): = \int_{0}^{x} \mu_{k}(t)\,dt\quad (k = 1, \ldots,i_{0}), \end{aligned}$$

8 $$\begin{aligned}& V_{l}(y): = \int_{0}^{y} \nu_{l}(t)\,dt\quad (l = 1, \ldots,j_{0}), \end{aligned}$$

9 $$\begin{aligned}& U(x): = \bigl(U_{1}(x),\ldots,U_{i_{0}}(x) \bigr),\quad\quad V(y): = \bigl(V_{1}(y),\ldots,V_{j_{0}}(y) \bigr) \quad (x,y \ge0). \end{aligned}$$

It follows that $U_{k}(m) = U_{m}^{(k)}$ ($k = 1,\ldots,i_{0}$; $m \in \mathbb{N}$), $V_{l}(n) = V_{n}^{(l)}$ ($l = 1,\ldots,j_{0}$; $n \in \mathbb{N}$), and for $x \in(m - 1,m)$, $U_{k}'(x) = \mu_{k}(x) = \mu_{m}^{(k)}$ ($k = 1,\ldots,i_{0}$; $m \in\mathbb{N}$); for $y \in(n - 1,n)$, $V_{l}'(y) = \nu_{l}(y) = \nu_{n}^{(l)}$ ($l = 1, \ldots,j_{0}$; $n \in\mathbb{N}$).

### Lemma 1

cf. [21]

*Suppose that* $g(t)$ (>0) *is decreasing in* $\mathbb{R}_{ +}$ *and strictly decreasing in* $[n_{0},\infty)$ ($n_{0} \in \mathbb{N}$), *satisfying* $\int_{0}^{\infty} g(t)\,dt \in\mathbb{R}_{ +}$. *We have*

10 $$ \int_{1}^{\infty} g(t)\,dt < \sum _{n = 1}^{\infty} g(n) < \int_{0}^{\infty} g(t)\,dt. $$

### Lemma 2

*If* $i_{0} \in\mathbb{N}$, $\alpha,M > 0$, $\Psi(u)$ *is a non*-*negative measurable function in* $(0,1]$, *and*

11 $$ D_{M}: = \Biggl\{ x \in\mathbb{R}_{ +}^{i_{0}};u = \sum_{i = 1}^{i_{0}} \biggl( \frac{x_{i}}{M} \biggr)^{\alpha} \le1 \Biggr\} , $$

*then we have the following transfer formula* (*cf*. [26]):

12 $$ \int\cdots \int_{D_{M}} \Psi \Biggl( \sum_{i = 1}^{i_{0}} \biggl( \frac{x_{i}}{M} \biggr)^{\alpha} \Biggr)\,dx_{1} \cdots dx_{s} = \frac{M^{i_{0}}\Gamma^{i_{0}} ( \frac{1}{\alpha} )}{\alpha^{i_{0}}\Gamma ( \frac{i_{0}}{\alpha} )} \int_{0}^{1} \Psi(u)u^{\frac{i_{0}}{\alpha} - 1}\,du. $$

### Lemma 3

*For* $i_{0},j_{0} \in\mathbb{N}$, $\mu_{m}^{(k)} \ge \mu_{m + 1}^{(k)}$ ($m \in\mathbb{N}$, $k = 1,\ldots,i_{0}$), $\nu_{n}^{(l)} \ge\nu_{n + 1}^{(l)}$ ($n \in\mathbb{N}$; $l = 1,\ldots,j_{0}$), $\alpha,\beta> 0$, $\varepsilon > 0$, *we have*

13 $$\begin{aligned}& \sum_{m} \Vert U_{m} \Vert _{\alpha}^{ - i_{0} - \varepsilon} \prod_{k = 1}^{i_{0}} \mu_{m}^{(k)} \le\frac{\Gamma^{i_{0}} ( \frac{1}{\alpha} )}{\varepsilon i_{0}^{\varepsilon/\alpha} \alpha^{i_{0} - 1}\Gamma ( \frac{i_{0}}{\alpha} )} + O(1), \end{aligned}$$

14 $$\begin{aligned}& \sum_{n} \Vert V_{n} \Vert _{\beta}^{ - j_{0} - \varepsilon} \prod_{k = 1}^{j_{0}} \nu_{n}^{(k)} \le\frac{\Gamma^{j_{0}} ( \frac{1}{\beta} )}{\varepsilon j_{0}^{\varepsilon/\beta} \beta^{j_{0} - 1}\Gamma ( \frac{j_{0}}{\beta} )} + \tilde{O}(1) \quad \bigl( \varepsilon\to0^{ +} \bigr). \end{aligned}$$

### Proof

For $M > i_{0}^{1/\alpha}$, we set

$$\Psi(u) = \textstyle\begin{cases} 0,&0 < u < \frac{i_{0}}{M^{\alpha}}, \\ \frac{1}{(Mu^{1/\alpha} )^{i_{0} + \varepsilon}},& \frac{i_{0}}{M^{\alpha}} \le u \le1. \end{cases} $$

By (12), it follows that

$$\begin{aligned} \int_{\{ x \in\mathbb{R}_{ +}^{i_{0}};x_{i} \ge1\}} \frac{dx}{ \Vert x \Vert _{\alpha}^{i_{0} + \varepsilon}} & = \lim_{M \to\infty} \int\cdots \int_{D_{M}} \Psi \Biggl( \sum_{i = 1}^{i_{0}} \biggl( \frac{x_{i}}{M} \biggr)^{\alpha} \Biggr) \,dx_{1} \cdots dx_{i_{0}} \\ &= \lim_{M \to\infty} \frac{M^{i_{0}}\Gamma^{i_{0}} ( \frac{1}{\alpha} )}{\alpha^{i_{0}}\Gamma ( \frac{i_{0}}{\alpha} )} \int_{i_{0}/M^{\alpha}}^{1} \frac{u^{\frac{i_{0}}{\alpha} - 1}}{(Mu^{1/\alpha} )^{i_{0} + \varepsilon}} \,du = \frac{\Gamma^{i_{0}} ( \frac{1}{\alpha} )}{\varepsilon i_{0}^{\varepsilon/\alpha} \alpha^{i_{0} - 1}\Gamma ( \frac{i_{0}}{\alpha} )}. \end{aligned}$$

Then by (10) and the above result, we find

$$\begin{aligned} 0 &< \sum_{\{ m \in\mathbb{N}^{i_{0}};m_{i} \ge2\}} \Vert U_{m} \Vert _{\alpha}^{ - i_{0} - \varepsilon} \prod_{k = 1}^{i_{0}} \mu_{m}^{(k)} \\ &= \sum_{\{ m \in\mathbb{N}^{i_{0}};m_{i} \ge2\}} \int_{\{ x \in \mathbb{N}^{i_{0}};m - 1 \le x_{i} < m\}} \bigl\Vert U(m) \bigr\Vert _{\alpha}^{ - i_{0} - \varepsilon} \prod_{k = 1}^{i_{0}} \mu_{m}^{(k)} \,dx \\ &< \sum_{\{ m \in\mathbb{N}^{i_{0}};m_{i} \ge2\}} \int_{\{ x \in \mathbb{N}^{i_{0}};m - 1 \le x_{i} < m\}} \bigl\Vert U(x) \bigr\Vert _{\alpha}^{ - i_{0} - \varepsilon} \prod_{k = 1}^{i_{0}} \mu_{m}^{(k)}(x) \,dx \\ &= \int_{\{ x \in\mathbb{N}^{i_{0}};x_{i} \ge1\}} \bigl\Vert U(x) \bigr\Vert _{\alpha}^{ - i_{0} - \varepsilon} \prod_{k = 1}^{i_{0}} \mu^{(k)}(x)\,dx \mathop{ =} \limits ^{\nu= U(x)} \int_{\{ \nu\in \mathbb{R}_{ +}^{i_{0}};\nu_{i} \ge\mu_{1}^{(i)}\}} \Vert \nu \Vert _{\alpha}^{ - i_{0} - \varepsilon} \,d \nu \\ &= \int_{\{ \nu\in\mathbb{R}_{ +}^{i_{0}};\nu_{i} \ge1\}} \Vert \nu \Vert _{\alpha}^{ - i_{0} - \varepsilon} \,d \nu+ O_{i_{0}}(1) = \frac{\Gamma^{i_{0}} ( \frac{1}{\alpha} )}{\varepsilon i_{0}^{\varepsilon/\alpha} \alpha^{i_{0} - 1}\Gamma ( \frac{i_{0}}{\alpha} )} + O_{i_{0}}(1). \end{aligned}$$

For $i_{0} = 1$, $0 < \sum_{\{ m \in\mathbb{N}^{i_{0}};m_{i} = 1\}} \Vert U_{m} \Vert _{\alpha}^{ - i_{0} - \varepsilon} \prod_{k = 1}^{i_{0}} \mu_{m}^{(k)} < \infty$; for $i_{0} \ge2$, $\mu^{(i)} = \max_{m}\mu_{m}^{(i)}$, $b = \sum_{i = 1}^{i_{0}} \mu^{(i)}$, in the same way, we find

$$\begin{aligned} 0 &< \sum_{\{ m \in\mathbb{N}^{i_{0}};\exists i,m_{i} = 1\}} \Vert U_{m} \Vert _{\alpha}^{ - i_{0} - \varepsilon} \prod_{k = 1}^{i_{0}} \mu_{m}^{(k)} \\ &\le \Vert U_{1} \Vert _{\alpha}^{ - i_{0} - \varepsilon} \prod_{k = 1}^{i_{0}} \mu_{1}^{(k)} + \sum_{i = 1}^{i_{0}} \mu^{(i)} \sum_{\{ m \in\mathbb{N}^{i_{0} - 1};m_{j} \ge2(j \ne i)\}} \Vert U_{m} \Vert _{\alpha}^{ - (i_{0} - 1) - (\varepsilon+ 1)} \prod_{k = 1(k \ne i)}^{i_{0}} \mu_{m}^{(k)} \\ &= O_{1}(1) + \frac{b\Gamma^{i_{0} - 1} ( \frac{1}{\alpha} )}{(1 + \varepsilon)(i_{0} - 1)^{(1 + \varepsilon)/\alpha} \alpha^{i_{0} - 2}\Gamma ( \frac{i_{0} - 1}{\alpha} )} + bO_{i_{0} - 1}(1) < \infty. \end{aligned}$$

Then we have

$$\begin{aligned} \sum_{m} \Vert U_{m} \Vert _{\alpha}^{ - i_{0} - \varepsilon} \prod_{k = 1}^{i_{0}} \mu_{m}^{(k)}& = \sum_{\{ m \in\mathbb{N}^{i_{0}};\exists i,m_{i} = 1\}} \sum _{m} \Vert U_{m} \Vert _{\alpha}^{ - i_{0} - \varepsilon} \prod_{k = 1}^{i_{0}} \mu_{m}^{(k)} \\ &\quad{} + \sum_{\{ m \in\mathbb{N}^{i_{0}};m_{j} \ge2\}} \Vert U_{m} \Vert _{\alpha}^{ - i_{0} - \varepsilon} \prod_{k = 1}^{i_{0}} \mu_{m}^{(k)} \\ &\le\frac{\Gamma^{i_{0}} ( \frac{1}{\alpha} )}{\varepsilon i_{0}^{\varepsilon/\alpha} \alpha^{i_{0} - 1}\Gamma ( \frac{i_{0}}{\alpha} )} + O(1) \quad \bigl(\varepsilon \to0^{ +} \bigr). \end{aligned}$$

Hence, we have (13). In the same way, we have (14). □

### Definition 1

For $\alpha,\beta> 0$, $0 < \lambda_{1} + \eta \le i_{0}$, $0 < \lambda_{2} + \eta\le j_{0}$, $\lambda_{1} + \lambda_{2} = \lambda$, we define two weight coefficients $w(\lambda_{1},n)$ and $W(\lambda_{2},m)$ as follows:

15 $$\begin{aligned}& w(\lambda_{1},n): = \sum_{m} \frac{(\min\{ \Vert U_{m} \Vert _{\alpha}, \Vert V_{n} \Vert _{\beta} \} )^{\eta}}{ (\max\{ \Vert U_{m} \Vert _{\alpha}, \Vert V_{n} \Vert _{\beta} \} )^{\lambda+ \eta}} \frac{ \Vert V_{n} \Vert _{\beta}^{\lambda_{2}}}{ \Vert U_{m} \Vert _{\alpha }^{i_{0} - \lambda_{1}}}\prod_{k = 1}^{i_{0}} \mu_{m}^{(k)}, \end{aligned}$$

16 $$\begin{aligned}& W(\lambda_{2},m): = \sum_{n} \frac{(\min\{ \Vert U_{m} \Vert _{\alpha}, \Vert V_{n} \Vert _{\beta} \} )^{\eta}}{ (\max\{ \Vert U_{m} \Vert _{\alpha}, \Vert V_{n} \Vert _{\beta} \} )^{\lambda+ \eta}} \frac{ \Vert U_{m} \Vert _{\alpha}^{\lambda_{1}}}{ \Vert V_{n} \Vert _{\beta }^{j_{0} - \lambda_{2}}}\prod_{l = 1}^{j_{0}} \nu_{n}^{(l)}. \end{aligned}$$

### Example 1

With regard to the assumptions of Definition 1, we set

$$k_{\lambda} (x,y) = \frac{(\min\{ x,y\} )^{\eta}}{(\max\{ x,y\} )^{\lambda+ \eta}} \quad (x,y > 0). $$

Then, (i) for fixed $y > 0$,

$$k_{\lambda} (x,y)\frac{1}{x^{i_{0} - \lambda_{1}}} = \textstyle\begin{cases} \frac{1}{y^{\lambda+ \eta} x^{i_{0} - \lambda_{1} - \eta}},&0 < x < y ,\\ \frac{y^{\eta}}{x^{i_{0} + \lambda_{2} + \eta}},&x \ge y, \end{cases} $$

is decreasing in $\mathbb{R}_{ +}$ and strictly decreasing in $([y] + 1,\infty)$. In the same way, for fixed $x > 0$, $k_{\lambda} (x,y)\frac{1}{y^{j_{0} - \lambda_{2}}}$ is decreasing in $\mathbb{R}_{ +}$ and strictly decreasing in $([x] + 1,\infty)$. We still have

17 $$ \begin{aligned}[b] k(\lambda_{1})&: = \int_{0}^{\infty} k_{\lambda} (u,1) \frac{du}{u^{1 - \lambda_{1}}} = \int_{0}^{\infty} \frac{(\min\{ u,1\} )^{\eta}}{(\max\{ u,1\} )^{\lambda+ \eta}} \frac{du}{u^{1 - \lambda_{1}}} \\ &= \int_{0}^{1} \frac{u^{\eta}}{u^{1 - \lambda_{1}}} \,du + \int_{1}^{\infty} \frac{1}{u^{\lambda+ \eta}} \frac{du}{u^{1 - \lambda _{1}}} = \frac{\lambda + 2\eta}{(\lambda_{1} + \eta)(\lambda_{2} + \eta)}. \end{aligned} $$

(ii) For $b > 0$, we have

$$\frac{d}{dx} \bigl(b + x^{\alpha} \bigr)^{\frac{1}{\alpha}} = \bigl(b + x^{\alpha} \bigr)^{\frac{1}{\alpha} - 1}x^{\alpha- 1} > 0\quad (x > 0). $$

Hence, for $m - 1 < x_{i} < m$ ($i = 1,\ldots,i_{0}$; $m \in\mathbb {N}$), we have $\Vert U(m) \Vert _{\alpha} > \Vert U(x) \Vert _{\alpha}$ and

$$\begin{aligned} &\frac{(\min\{ \Vert U(m) \Vert _{\alpha}, \Vert V_{n} \Vert _{\beta} \} )^{\eta}}{(\max\{ \Vert U(m) \Vert _{\alpha}, \Vert V_{n} \Vert _{\beta} \} )^{\lambda+ \eta}} \frac{1}{ \Vert U(m) \Vert _{\alpha}^{i_{0} - \lambda_{1}}} \\ &\quad < \frac{(\min\{ \Vert U(x) \Vert _{\alpha}, \Vert V_{n} \Vert _{\beta} \} )^{\eta}}{(\max\{ \Vert U(x) \Vert _{\alpha}, \Vert V_{n} \Vert _{\beta} \} )^{\lambda+ \eta}} \frac{1}{ \Vert U(x) \Vert _{\alpha}^{i_{0} - \lambda_{1}}}; \end{aligned} $$

for $m < x_{i} < m + 1$ ($i = 1,\ldots,i_{0}$; $m \in\mathbb{N}$), we have $\Vert U(m) \Vert _{\alpha} < \Vert U(x) \Vert _{\alpha}$ and

$$\begin{aligned} & \frac{(\min\{ \Vert U(m) \Vert _{\alpha}, \Vert V_{n} \Vert _{\beta} \} )^{\eta}}{(\max\{ \Vert U(m) \Vert _{\alpha}, \Vert V_{n} \Vert _{\beta} \} )^{\lambda+ \eta}} \frac{1}{ \Vert U(m) \Vert _{\alpha}^{i_{0} - \lambda_{1}}} \\ &\quad > \frac{(\min\{ \Vert U(x) \Vert _{\alpha}, \Vert V_{n} \Vert _{\beta} \} )^{\eta}}{(\max\{ \Vert U(x) \Vert _{\alpha}, \Vert V_{n} \Vert _{\beta} \} )^{\lambda+ \eta}} \frac{1}{ \Vert U(x) \Vert _{\alpha}^{i_{0} - \lambda_{1}}}. \end{aligned} $$

### Lemma 4

*With regard to the assumptions of Definition* 1, (i) *we have*

18 $$\begin{aligned}& w(\lambda_{1},n) < K_{2}(\lambda_{1}) \quad \bigl(n \in\mathbb{N}^{j_{0}} \bigr), \end{aligned}$$

19 $$\begin{aligned}& W(\lambda_{2},m) < K_{1}(\lambda_{1}) \quad \bigl(m \in\mathbb{N}^{i_{0}} \bigr), \end{aligned}$$

*where*

20 $$ K_{1}(\lambda_{1}) = \frac{\Gamma^{j_{0}} ( \frac{1}{\beta} )}{\beta^{j_{0} - 1}\Gamma ( \frac{j_{0}}{\beta} )}k( \lambda_{1}),\qquad K_{2}(\lambda_{1}) = \frac{\Gamma^{i_{0}} ( \frac{1}{\alpha} )}{\alpha^{i_{0} - 1}\Gamma ( \frac{i_{0}}{\alpha} )}k(\lambda_{1}); $$

(ii) *for* $\mu_{m}^{(k)} \ge \mu_{m + 1}^{(k)}$ ($m \in\mathbb{N}$), $\nu_{n}^{(l)} \ge\nu_{n + 1}^{(l)}$ ($n \in\mathbb{N}$), $U_{\infty}^{(k)} = V_{\infty}^{(l)}$ ($k = 1,\ldots,i_{0}$, $l = 1, \ldots,j_{0}$), $0 < \lambda_{1} + \eta\le i_{0}$, $\lambda_{2} + \eta> 0$, $0 < \varepsilon< p\lambda_{1}$ ($p > 1$), *we have*

21 $$ 0 < K_{2}(\lambda_{1}) \bigl(1 - \theta_{\lambda} (n) \bigr) < w(\lambda_{1},n) \quad \bigl(n \in\mathbb{N}^{j_{0}} \bigr), $$

*where*, *for* $c: = \max_{1 \le k \le i_{0}}\{ \mu_{1}^{(k)}\}$ (>0),

22 $$ \theta_{\lambda} (n): = \frac{1}{k(\lambda_{1})} \int_{0}^{ci_{0}^{1/\alpha} / \Vert V_{n} \Vert _{\beta}} \frac{(\min\{ v,1\} )^{\eta} v^{\lambda_{1} - 1}}{(\max\{ v,1\} )^{\lambda+ \eta}} \,dv = O \biggl( \frac{1}{ \Vert V_{n} \Vert _{\beta}^{\lambda_{1} + \eta}} \biggr). $$

### Proof

(i) By (10), (12) and Example 1(ii), for $0 < \lambda_{1} + \eta\le i_{0}$, $\lambda> 0$, it follows that

$$\begin{aligned} w(\lambda_{1},n) &= \sum_{m} \int_{\{ x \in\mathbb{N}^{i_{0}};m_{i} - 1 \le x_{i} \le m_{i}\}} \frac{(\min\{ \Vert U(m) \Vert _{\alpha}, \Vert V_{n} \Vert _{\beta} \} )^{\eta}}{(\max\{ \Vert U(m) \Vert _{\alpha}, \Vert V_{n} \Vert _{\beta} \} )^{\lambda+ \eta}} \\ &\quad{} \times\frac{ \Vert V_{n} \Vert _{\beta}^{\lambda_{2}}}{ \Vert U(m) \Vert _{\alpha}^{i_{0} - \lambda{}_{1}}}\prod_{k = 1}^{i_{0}} \mu_{m}^{(k)} \,dx \\ &< \sum_{m} \int_{\{ x \in\mathbb{N}^{i_{0}};m_{i} - 1 \le x_{i} \le m_{i}\}} \frac{(\min\{ \Vert U(x) \Vert _{\alpha}, \Vert V_{n} \Vert _{\beta} \} )^{\eta}}{(\max\{ \Vert U(x) \Vert _{\alpha}, \Vert V_{n} \Vert _{\beta} \} )^{\lambda+ \eta}} \\ &\quad{} \times\frac{ \Vert V_{n} \Vert _{\beta}^{\lambda_{2}}}{ \Vert U(x) \Vert _{\alpha}^{i_{0} - \lambda{}_{1}}}\prod_{k = 1}^{i_{0}} \mu_{m}^{(k)}(x) \,dx \\ & = \int_{\mathbb{R}_{ +}^{i_{0}}} \frac{(\min\{ \Vert U(x) \Vert _{\alpha}, \Vert V_{n} \Vert _{\beta} \} )^{\eta}}{ (\max\{ \Vert U(x) \Vert _{\alpha}, \Vert V{}_{n} \Vert _{\beta} \} )^{\lambda+ \eta}} \frac{ \Vert V_{n} \Vert _{\beta}^{\lambda_{2}}}{ \Vert U(x) \Vert _{\alpha }^{i_{0} - \lambda{}_{1}}}\prod _{k = 1}^{i_{0}} \mu^{(k)}(x) \,dx \\ & \mathop{\le}^{u = U(x)} \int_{\mathbb{R}_{ +}^{i_{0}}} \frac{(\min\{ \Vert u \Vert _{\alpha}, \Vert V_{n} \Vert _{\beta} \} )^{\eta}}{ (\max\{ \Vert u \Vert _{\alpha}, \Vert V{}_{n} \Vert _{\beta} \} )^{\lambda + \eta}} \frac{ \Vert V_{n} \Vert _{\beta}^{\lambda_{2}}}{ \Vert u \Vert _{\alpha}^{i_{0} - \lambda{}_{1}}}\,du \\ & = \lim_{M \to\infty} \int_{D_{M}} \frac{(\min\{ M [ \sum_{i = 1}^{i_{0}} ( \frac{u_{i}}{M} )^{\alpha} ]^{1/\alpha}, \Vert V_{n} \Vert _{\beta} \} )^{\eta}}{(\max\{ M [ \sum_{i = 1}^{i_{0}} ( \frac{u_{i}}{M} )^{\alpha} ]^{1/\alpha}, \Vert V{}_{n} \Vert _{\beta} \} )^{\lambda+ \eta }} \frac{M^{\lambda_{1} - i_{0}} \Vert V_{n} \Vert _{\beta}^{\lambda_{2}}\,du}{ [ \sum_{i = 1}^{i_{0}} ( \frac{u_{i}}{M} )^{\alpha} ]^{(i_{0} - \lambda_{1})/\alpha}} \\ & = \lim_{M \to\infty} \frac{M^{i_{0}}\Gamma^{i_{0}} ( \frac{1}{\alpha} )}{\alpha^{i_{0}}\Gamma ( \frac{i_{0}}{\alpha} )} \int_{0}^{1} \frac{(\min\{ Mu^{1/\alpha}, \Vert V_{n} \Vert _{\beta} \} )^{\eta} \Vert V_{n} \Vert _{\beta}^{\lambda_{2}}}{(\max\{ Mu^{1/\alpha}, \Vert V_{n} \Vert _{\beta} \} )^{\lambda+ \eta}} \frac{u^{\frac{i_{0}}{\alpha} - 1}\,du}{M^{i_{0} - \lambda _{1}}u^{(i_{0} - \lambda_{1})/\alpha}} \\ & = \lim_{M \to\infty} \frac{M^{i_{0}}\Gamma^{i_{0}} ( \frac{1}{\alpha} )}{\alpha^{i_{0}}\Gamma ( \frac{i_{0}}{\alpha} )} \int_{0}^{1} \frac{(\min\{ Mu^{1/\alpha}, \Vert V_{n} \Vert _{\beta} \} )^{\eta} \Vert V_{n} \Vert _{\beta}^{\lambda_{2}}}{(\max\{ Mu^{1/\alpha}, \Vert V_{n} \Vert _{\beta} \} )^{\lambda+ \eta}} u^{\frac{\lambda_{1}}{\alpha} - 1} \,du \\ & \mathop{=}^{v = \frac{Mu^{1/\alpha}}{ \Vert V_{n} \Vert _{\beta}}} \frac{\Gamma^{i_{0}} ( \frac{1}{\alpha} )}{\alpha^{i_{0} - 1}\Gamma ( \frac{i_{0}}{\alpha} )} \int_{0}^{\infty} \frac{(\min\{ v,1\} )^{\eta} v^{\lambda_{1} - 1}}{(\max \{ v,1\} )^{\lambda+ \eta}} \,dv \\ & = \frac{\Gamma^{i_{0}} ( \frac{1}{\alpha} )}{\alpha^{i_{0} - 1}\Gamma ( \frac{i_{0}}{\alpha} )} \frac{\lambda+ 2\eta}{ (\lambda_{1} + \eta)(\lambda_{2} + \eta)} = K_{2}( \lambda_{1}). \end{aligned}$$

Hence, we have (18). In the same way, we have (19).

(ii) By (10) and in the same way, for $c = \max_{1 \le k \le i_{0}}\{ \mu_{1}^{(k)}\}$ (>0), we have

$$\begin{aligned} w(\lambda_{1},n) &\ge\sum_{m} \int_{\{ x \in\mathbb{N}^{i_{0}};m_{i} \le x_{i} \le m_{i} + 1\}} \frac{(\min\{ \Vert U(m) \Vert _{\alpha}, \Vert V_{n} \Vert _{\beta} \} )^{\eta}}{ (\max\{ \Vert U(m) \Vert _{\alpha}, \Vert V_{n} \Vert _{\beta} \} )^{\lambda + \eta}} \\ &\quad{} \times\frac{ \Vert V_{n} \Vert _{\beta}^{\lambda_{2}}}{ \Vert U(m) \Vert _{\alpha}^{i_{0} - \lambda{}_{1}}}\prod_{k = 1}^{i_{0}} \mu_{m + 1}^{(k)} \,dx \\ & > \sum_{m} \int_{\{ x \in\mathbb{N}^{i_{0}};m_{i} \le x_{i} \le m_{i} + 1\}} \frac{(\min\{ \Vert U(x) \Vert _{\alpha}, \Vert V_{n} \Vert _{\beta} \} )^{\eta}}{(\max\{ \Vert U(x) \Vert _{\alpha}, \Vert V_{n} \Vert _{\beta} \} )^{\lambda+ \eta}} \\ &\quad{} \times\frac{ \Vert V_{n} \Vert _{\beta}^{\lambda_{2}}}{ \Vert U(x) \Vert _{\alpha}^{i_{0} - \lambda{}_{1}}}\prod_{k = 1}^{i_{0}} \mu^{(k)}(x) \,dx \\ & = \int_{[1,\infty)^{i_{0}}} \frac{(\min\{ \Vert U(x) \Vert _{\alpha}, \Vert V_{n} \Vert _{\beta} \} )^{\eta}}{ (\max\{ \Vert U(x) \Vert _{\alpha}, \Vert V{}_{n} \Vert _{\beta} \} )^{\lambda+ \eta}} \frac{ \Vert V_{n} \Vert _{\beta}^{\lambda_{2}}}{ \Vert U(x) \Vert _{\alpha }^{i_{0} - \lambda{}_{1}}}\prod _{k = 1}^{i_{0}} \mu^{(k)}(x) \,dx \\ & \mathop{\ge}^{v = U(x)} \int_{[c,\infty)^{i_{0}}} \frac{(\min\{ \Vert v \Vert _{\alpha}, \Vert V_{n} \Vert _{\beta} \} )^{\eta}}{ (\max\{ \Vert v \Vert _{\alpha}, \Vert V{}_{n} \Vert _{\beta} \} )^{\lambda + \eta}} \frac{ \Vert V_{n} \Vert _{\beta}^{\lambda_{2}}}{ \Vert v \Vert _{\alpha}^{i_{0} - \lambda{}_{1}}}\,dv. \end{aligned}$$

For $M > ci_{0}^{1/\alpha}$, we set

$$\Psi(u) = \textstyle\begin{cases} 0,& 0 < u < \frac{c^{\alpha} i_{0}}{M^{\alpha}}, \\ \frac{(\min\{ Mu^{1/\alpha}, \Vert V_{n} \Vert _{\beta} \} )^{\eta}}{(\max\{ Mu^{1/\alpha}, \Vert V_{n} \Vert _{\beta} \} )^{\lambda+ \eta}} \frac{ \Vert V_{n} \Vert _{\beta}^{\lambda _{2}}}{(Mu^{1/\alpha} )^{i_{0} - \lambda_{1}}}, &\frac{c^{\alpha} i_{0}}{M^{\alpha}} \le u \le1. \end{cases} $$

By (12), it follows that

$$\begin{aligned}& \int_{\{ x \in\mathbb{R}_{ +}^{i_{0}};x_{i} \ge c\}} \frac{(\min\{ \Vert x \Vert _{\alpha}, \Vert V_{n} \Vert _{\beta} \} )^{\eta}}{ (\max\{ \Vert x \Vert _{\alpha}, \Vert V{}_{n} \Vert _{\beta} \} )^{\lambda+ \eta}} \frac{ \Vert V_{n} \Vert _{\beta}^{\lambda_{2}}}{ \Vert x \Vert _{\alpha}^{i_{0} - \lambda{}_{1}}}\,dx \\& \quad = \lim_{M \to\infty} \int\cdots \int_{D_{M}} \Psi \Biggl( \sum_{i = 1}^{i_{0}} \biggl( \frac{x_{i}}{M} \biggr)^{\alpha} \Biggr) \,dx_{1} \cdots dx_{i_{0}} \\& \quad = \lim_{M \to\infty} \frac{M^{i_{0}}\Gamma^{i_{0}} ( \frac{1}{\alpha} )}{\alpha^{i_{0}}\Gamma ( \frac{i_{0}}{\alpha} )} \int_{c^{\alpha} i_{0}/M^{\alpha}}^{1} \frac{(\min\{ Mu^{\frac{1}{\alpha}}, \Vert V_{n} \Vert _{\beta} \} )^{\eta}}{ (\max\{ Mu^{\frac{1}{\alpha}}, \Vert V_{n} \Vert _{\beta} \} )^{\lambda+ \eta}} \frac{ \Vert V_{n} \Vert _{\beta}^{\lambda_{2}}u^{\frac {i_{0}}{\alpha} - 1}\,du}{(Mu^{\frac{1}{\alpha}} )^{i_{0} - \lambda_{1}}} \\& \quad \mathop{ =} \limits ^{v = \frac{Mu^{1/\alpha}}{ \Vert V_{n} \Vert _{\beta}}} \frac{\Gamma^{i_{0}} ( \frac{1}{\alpha} )}{\alpha^{i_{0} - 1}\Gamma ( \frac{i_{0}}{\alpha} )} \int_{ci_{0}^{1/\alpha} / \Vert V_{n} \Vert _{\beta}}^{1} \frac {(\min\{ v,1\} )^{\eta} v^{\lambda_{1} - 1}}{(\max\{ v,1\} )^{\lambda+ \eta}} \,dv. \end{aligned}$$

Hence, we have

$$\begin{aligned} w(\lambda_{1},n) &> \frac{\Gamma^{i_{0}} ( \frac{1}{\alpha} )}{\alpha^{i_{0} - 1}\Gamma ( \frac{i_{0}}{\alpha} )} \int_{ci_{0}^{1/\alpha} / \Vert V_{n} \Vert _{\beta}}^{1} \frac {(\min\{ v,1\} )^{\eta} v^{\lambda_{1} - 1}}{(\max\{ v,1\} )^{\lambda+ \eta}} \,dv \\ & = K_{2}(\lambda_{1}) \bigl(1 - \theta_{\lambda} (n) \bigr) > 0. \end{aligned}$$

For $\Vert V_{n} \Vert _{\beta} \ge ci_{0}^{1/\alpha}$, we obtain

$$\begin{aligned} 0 &< \theta_{\lambda} (n) = \frac{1}{k(\lambda_{1})} \int_{0}^{ci_{0}^{1/\alpha} / \Vert V_{n} \Vert _{\beta}} \frac{(\min\{ v,1\} )^{\eta} v^{\lambda_{1} - 1}}{(\max \{ v,1\} )^{\lambda+ \eta}} \,dv \\ & = \frac{1}{k(\lambda_{1})} \int_{0}^{ci_{0}^{1/\alpha} / \Vert V_{n} \Vert _{\beta}} v^{\lambda_{1} + \eta- 1} \,dv = \frac{1}{(\lambda_{1} + \eta)k(\lambda_{1})} \biggl( \frac{ci_{0}^{1/\alpha}}{ \Vert V{}_{n} \Vert _{\beta}} \biggr)^{\lambda_{1} + \eta}, \end{aligned}$$

and then (21) and (22) follow. □

## Main results

Setting functions

$$\begin{aligned}& \Phi(m): = \frac{ \Vert U_{m} \Vert _{\alpha}^{p(i_{0} - \lambda _{1}) - i_{0}}}{(\prod_{k = 1}^{i_{0}} \mu_{m}^{(k)})^{p - 1}} \quad \bigl(m \in \mathbb{N}^{i_{0}} \bigr), \\& \Psi(n): = \frac{ \Vert V_{n} \Vert _{\beta}^{q(j_{0} - \lambda _{2}) - j_{0}}}{(\prod_{l = 1}^{j_{0}} \nu_{n}^{(l)})^{q - 1}} \quad \bigl(n \in \mathbb{N}^{j_{0}} \bigr), \end{aligned}$$

and the following normed spaces:

$$\begin{aligned}& l_{p,\Phi}: = \biggl\{ a = \{ a_{m}\}; \Vert a \Vert _{p,\Phi}: = \biggl\{ \sum_{m} \Phi(m) \vert a_{m} \vert ^{p} \biggr\} ^{\frac{1}{p}} < \infty \biggr\} , \\& l_{q,\Psi}: = \biggl\{ b = \{ b_{n}\}; \Vert b \Vert _{q,\Psi}: = \biggl\{ \sum_{n} \Psi(n) \vert b_{n} \vert ^{q} \biggr\} ^{\frac{1}{q}} < \infty \biggr\} , \\& l_{p,\Psi^{1 - p}}: = \biggl\{ c = \{ c_{n}\}; \Vert c \Vert _{p,\Psi^{1 - p}}: = \biggl\{ \sum_{n} \Psi^{1 - p}(n) \vert c_{n} \vert ^{p} \biggr\} ^{\frac{1}{p}} < \infty \biggr\} , \end{aligned}$$

we have the following.

### Theorem 1

*If* $p > 1$, $\frac{1}{p} + \frac{1}{q} = 1$, $\alpha ,\beta> 0$, $\lambda> 0$, $0 < \lambda_{1} + \eta\le i_{0}$, $0 < \lambda_{2} + \eta\le j_{0}$, $\lambda_{1} + \lambda_{2} = \lambda$, *then for* $a_{m},b_{n} \ge0$, $a = \{ a_{m}\} \in l_{p, \Phi}$, $b = \{ b_{n}\} \in l_{q, \Psi}$, $\Vert a \Vert _{p, \Phi}, \Vert b \Vert _{q,\Psi} > 0$, *we have the following equivalent inequalities*:

23 $$\begin{aligned}& I: = \sum_{n} \sum_{m} \frac{(\min\{ \Vert U_{m} \Vert _{\alpha}, \Vert V_{n} \Vert _{\beta} \} )^{\eta}}{ (\max\{ \Vert U_{m} \Vert _{\alpha}, \Vert V_{n} \Vert _{\beta} \} )^{\lambda+ \eta}} a_{m}b_{n} < K_{1}^{\frac{1}{p}}( \lambda_{1})K_{2}^{\frac{1}{q}}(\lambda_{1}) \Vert a \Vert _{p,\Phi} \Vert b \Vert _{q,\Psi}, \end{aligned}$$

24 $$\begin{aligned}& \begin{aligned}[b] J&: = \biggl\{ \sum_{n} \frac{\prod_{k = 1}^{j_{0}} v_{n}^{(k)}}{ \Vert V_{n} \Vert _{\beta}^{j_{0} - p\lambda_{2}}} \biggl[ \sum_{m} \frac{(\min \{ \Vert U_{m} \Vert _{\alpha}, \Vert V_{n} \Vert _{\beta} \} )^{\eta} a_{m}}{(\max\{ \Vert U_{m} \Vert _{\alpha}, \Vert V_{n} \Vert _{\beta} \} )^{\lambda+ \eta}} \biggr]^{p} \biggr\} ^{\frac{1}{p}} \\ & < K_{1}^{\frac{1}{p}}(\lambda_{1})K_{2}^{\frac{1}{q}}( \lambda_{1}) \Vert a \Vert _{p,\Phi}, \end{aligned} \end{aligned}$$

*where*

25 $$ K_{1}^{\frac{1}{p}}(\lambda_{1})K_{2}^{\frac{1}{q}}( \lambda_{1}) = \biggl[ \frac{\Gamma^{j_{0}} ( \frac{1}{\beta} )}{\beta ^{j_{0} - 1}\Gamma ( \frac{j_{0}}{\beta} )} \biggr]^{\frac{1}{p}} \biggl[ \frac{\Gamma^{i_{0}} ( \frac{1}{\alpha} )}{\alpha^{i_{0} - 1}\Gamma ( \frac{i_{0}}{\alpha} )} \biggr]^{\frac{1}{q}}k(\lambda _{1}). $$

### Proof

By Hölder’s inequality with weight (cf. [27]), we have

$$\begin{aligned} I &= \sum_{n} \sum_{m} \frac{(\min\{ \Vert U_{m} \Vert _{\alpha}, \Vert V_{n} \Vert _{\beta} \} )^{\eta}}{ (\max\{ \Vert U_{m} \Vert _{\alpha}, \Vert V_{n} \Vert _{\beta} \} )^{\lambda+ \eta}} \\ &\quad{} \times \biggl[ \frac{ \Vert U_{m} \Vert _{\alpha}^{\frac{i_{0} - \lambda_{1}}{q}}}{ \Vert V_{n} \Vert _{\beta}^{\frac{j_{0} - \lambda_{2}}{p}}}\frac{(\prod_{l = 1}^{j_{0}} \nu_{n}^{(l)} )^{\frac{1}{p}}a_{m}}{(\prod_{k = 1}^{i_{0}} \mu_{m}^{(k)} )^{\frac {1}{q}}} \biggr] \biggl[ \frac{ \Vert V_{n} \Vert _{\beta}^{\frac{j_{0} - \lambda_{2}}{p}}}{ \Vert U_{m} \Vert _{\alpha}^{\frac{i_{0} - \lambda_{1}}{q}}}\frac{(\prod_{k = 1}^{i_{0}} \mu_{m}^{(k)} )^{\frac{1}{q}}b_{n}}{(\prod_{l = 1}^{j_{0}} \nu_{n}^{(l)} )^{\frac {1}{p}}} \biggr] \\ & \le \biggl[ \sum_{m} W(\lambda_{2},m) \frac{ \Vert U_{m} \Vert _{\alpha}^{p(i_{0} - \lambda_{1}) - i_{0}}a_{m}^{p}}{(\prod_{k = 1}^{i_{0}} \mu_{m}^{(k)} )^{p - 1}} \biggr]^{\frac{1}{p}} \biggl[ \sum _{n} w(\lambda_{1},n) \frac{ \Vert V_{n} \Vert _{\beta}^{q(j_{0} - \lambda_{2}) - j_{0}}b_{n}^{q}}{(\prod_{l = 1}^{j_{0}} \nu_{n}^{(l)} )^{q - 1}} \biggr]^{\frac{1}{q}}. \end{aligned}$$

Then by (18) and (19), we have (23). We set

$$b_{n}: = \frac{\prod_{l = 1}^{j_{0}} \nu_{n}^{(l)}}{ \Vert V_{n} \Vert _{\beta}^{j_{0} - p\lambda_{2}}} \biggl[ \sum_{m} \frac{(\min\{ \Vert U_{m} \Vert _{\alpha}, \Vert V_{n} \Vert _{\beta} \} )^{\eta} a_{m}}{(\max\{ \Vert U_{m} \Vert _{\alpha}, \Vert V_{n} \Vert _{\beta} \} )^{\lambda+ \eta}} \biggr]^{p - 1},\quad n \in\mathbb {N}^{j_{0}}. $$

Then we have $J = \Vert b \Vert _{q,\Psi}^{q - 1}$. Since the right-hand side of (24) is finite, it follows $J < \infty$. If $J = 0$, then (24) is trivially valid; if $J > 0$, then by (23), we have

$$\begin{aligned}& \Vert b \Vert _{q,\Psi}^{q} = J^{p} = I < K_{1}^{\frac{1}{p}}(\lambda_{1})K_{2}^{\frac{1}{q}}( \lambda_{1}) \Vert a \Vert _{p,\Phi} \Vert b \Vert _{q,\Psi}, \\& \Vert b \Vert _{q,\Psi}^{q - 1} = J < K_{1}^{\frac{1}{p}}( \lambda_{1})K_{2}^{\frac{1}{q}}(\lambda_{1}) \Vert a \Vert _{p,\Phi}, \end{aligned}$$

namely (24) follows.

On the other hand, assuming that (24) is valid, by Hölder’s inequality (cf. [27]), we have

26 $$ \begin{aligned}[b] I &= \sum_{n} \frac{(\prod_{l = 1}^{j_{0}} v_{n}^{(l)} )^{1/p}}{ \Vert V_{n} \Vert _{\beta}^{(j_{0}/p) - \lambda_{2}}} \sum_{m} \frac{(\min\{ \Vert U_{m} \Vert _{\alpha}, \Vert V_{n} \Vert _{\beta} \} )^{\eta}}{ (\max\{ \Vert U_{m} \Vert _{\alpha}, \Vert V_{n} \Vert _{\beta} \} )^{\lambda+ \eta}} a_{m} \\ &\quad{} \times\frac{ \Vert V_{n} \Vert _{\beta}^{(j_{0}/p) - \lambda_{2}}}{(\prod_{l = 1}^{j_{0}} \nu_{n}^{(l)})^{1/p}} b_{n} \le J \Vert b \Vert _{q,\Psi}. \end{aligned} $$

Then by (24) we have (23), which is equivalent to (24). □

### Theorem 2

*With regard to the assumptions of Theorem* 1, *if* $\mu_{m}^{(k)} \ge\mu_{m + 1}^{(k)}$ ($m \in\mathbb{N}$), $\nu _{n}^{(l)} \ge \nu_{n + 1}^{(l)}$ ($n \in\mathbb{N}$), $U_{\infty}^{(k)} = V_{\infty}^{(l)} = \infty$ ($k = 1,\ldots,i_{0}$, $l = 1,\ldots,j_{0}$), *then the constant factor* $K_{1}^{\frac{1}{p}}(\lambda_{1})K_{2}^{\frac{1}{q}}(\lambda_{1})$ *in* (23) *and* (24) *is the best possible*.

### Proof

For $0 < \varepsilon< p(\lambda_{1} + \eta )$, $\tilde{\lambda}_{1} = \lambda_{1} - \frac{\varepsilon}{p}$ ($\in ( - \eta, - \eta+ i_{0})$), $\tilde{\lambda}_{2} = \lambda_{2} + \frac {\varepsilon}{ p}$ ($> - \eta$), we set

$$\begin{aligned}& \tilde{a} = \{ \tilde{a}_{m}\},\quad\quad \tilde{a}_{m}: = \Vert U_{m} \Vert _{\alpha}^{ - i_{0} + \tilde{\lambda}_{1}}\prod _{k = 1}^{i_{0}} \mu_{m}^{(k)}\quad \bigl(m \in\mathbb{N}^{i_{0}} \bigr), \\& \tilde{b} = \{ \tilde{b}_{n}\},\quad\quad \tilde{b}_{n}: = \Vert V_{n} \Vert _{\beta}^{ - j_{0} + \tilde{\lambda}_{2} - \varepsilon} \prod _{l = 1}^{j_{0}} \nu_{n}^{(l)}\quad \bigl(n \in\mathbb{N}^{j_{0}} \bigr). \end{aligned}$$

Then by (13) and (14), we obtain

$$\begin{aligned} \Vert \tilde{a} \Vert _{p,\Phi} \Vert \tilde{b} \Vert _{q,\Psi} &= \biggl[ \sum_{m} \frac{ \Vert U_{m} \Vert _{\alpha}^{p(i_{0} - \lambda_{1}) - i_{0}}\tilde{a}_{m}^{p}}{(\prod_{k = 1}^{i_{0}} \mu_{m}^{(k)} )^{p - 1}} \biggr]^{\frac{1}{p}} \biggl[ \sum _{n} \frac{ \Vert V_{n} \Vert _{\beta}^{q(j_{0} - \lambda_{2}) - j_{0}}\tilde{b}_{n}^{q}}{(\prod_{l = 1}^{j_{0}} \nu_{n}^{(l)} )^{q - 1}} \biggr]^{\frac{1}{q}} \\ & = \Biggl( \sum_{m} \Vert U_{m} \Vert _{\alpha}^{ - i_{0} - \varepsilon} \prod_{k = 1}^{i_{0}} \mu_{m}^{(k)} \Biggr)^{\frac{1}{p}} \Biggl( \sum _{n} \Vert V_{n} \Vert _{\beta}^{ - j_{0} - \varepsilon} \prod_{l = 1}^{j_{0}} \nu_{n}^{(l)} \Biggr)^{\frac{1}{q}} \\ & \le\frac{1}{\varepsilon} \biggl( \frac{\Gamma^{i_{0}} ( \frac{1}{\alpha} )}{i_{0}^{\varepsilon/\alpha} \alpha^{i_{0} - 1}\Gamma ( \frac{i_{0}}{\alpha} )} + \varepsilon O(1) \biggr)^{\frac{1}{p}} \biggl( \frac{\Gamma^{j_{0}} ( \frac{1}{\beta} )}{j_{0}^{\varepsilon/\beta} \beta^{j_{0} - 1}\Gamma ( \frac{j_{0}}{\beta} )} + \varepsilon\tilde{O}(1) \biggr)^{\frac{1}{q}}. \end{aligned}$$

By (21) and (22), we find

$$\begin{aligned} \tilde{I}&: = \sum_{n} \biggl[ \sum _{m} \frac{(\min\{ \Vert U_{m} \Vert _{\alpha}, \Vert V_{n} \Vert _{\beta} \} )^{\eta}}{ (\max\{ \Vert U_{m} \Vert _{\alpha}, \Vert V_{n} \Vert _{\beta} \} )^{\lambda+ \eta}} \tilde{a}_{m} \biggr] \tilde{b}_{n} \\ & = \sum_{n} w(\tilde{\lambda}_{1},n) \Vert V_{n} \Vert _{\beta}^{ - j_{0} - \varepsilon} \prod _{l = 1}^{j_{0}} \nu_{n}^{(l)} \\ & > K_{2}(\tilde{\lambda}_{1})\sum _{n} \biggl( 1 - O \biggl( \frac{1}{ \Vert V_{n} \Vert _{\beta}^{\lambda_{1} + \eta}} \biggr) \biggr) \Vert V_{n} \Vert _{\beta}^{ - j_{0} - \varepsilon} \prod _{l = 1}^{j_{0}} \nu_{n}^{(l)} \\ & = K_{2}(\tilde{\lambda}_{1}) \biggl( \frac{\Gamma^{j_{0}} ( \frac{1}{\beta} )}{\varepsilon j_{0}^{\varepsilon/\beta} \beta^{j_{0} - 1}\Gamma ( \frac{j_{0}}{\beta} )} + \tilde{O}(1) - O_{1}(1) \biggr). \end{aligned}$$

If there exists a constant $K \le K_{1}^{\frac{1}{p}}(\lambda_{1})K_{2}^{\frac{1}{q}}(\lambda_{1})$ such that (23) is valid when replacing $K_{1}^{\frac{1}{p}}(\lambda_{1})K_{2}^{\frac{1}{q}}( \lambda_{1})$ by *K*, then we have $\varepsilon\tilde{I} < \varepsilon K \Vert \tilde{a} \Vert _{p,\Phi} \Vert \tilde{b} \Vert _{q,\Psi}$, namely

$$\begin{aligned}& K_{2} \biggl(\lambda_{1} - \frac{\varepsilon}{p} \biggr) \biggl( \frac{\Gamma^{j_{0}} ( \frac{1}{\beta} )}{j_{0}^{\varepsilon /\beta} \beta^{j_{0} - 1}\Gamma ( \frac{j_{0}}{\beta} )} + \varepsilon \tilde{O}(1) - \varepsilon O_{1}(1) \biggr) \\& \quad < K \biggl( \frac{\Gamma^{i_{0}} ( \frac{1}{\alpha} )}{i_{0}^{\varepsilon/\alpha} \alpha^{i_{0} - 1}\Gamma ( \frac{i_{0}}{\alpha} )} + \varepsilon O(1) \biggr)^{\frac{1}{p}} \biggl( \frac{\Gamma^{j_{0}} ( \frac{1}{\beta} )}{j_{0}^{\varepsilon/\beta} \beta^{j_{0} - 1}\Gamma ( \frac{j_{0}}{\beta} )} + \varepsilon\tilde{O}(1) \biggr)^{\frac{1}{q}}. \end{aligned}$$

For $\varepsilon\to0^{ +}$, we find

$$\frac{\Gamma^{j_{0}} ( \frac{1}{\beta} )}{\beta^{j_{0} - 1}\Gamma ( \frac{j_{0}}{\beta} )} \frac{\Gamma^{i_{0}} ( \frac{1}{\alpha} )k(\lambda_{1})}{\alpha^{i_{0} - 1}\Gamma ( \frac{i_{0}}{\alpha} )} \le K \biggl[ \frac{\Gamma^{i_{0}} ( \frac{1}{\alpha} )}{\alpha^{i_{0} - 1}\Gamma ( \frac{i_{0}}{\alpha} )} \biggr]^{\frac{1}{p}} \biggl[ \frac{\Gamma^{j_{0}} ( \frac{1}{\beta} )}{\beta^{j_{0} - 1}\Gamma ( \frac{j_{0}}{\beta} )} \biggr]^{\frac{1}{q}}, $$

and then $K_{1}^{\frac{1}{p}}(\lambda_{1})K_{2}^{\frac{1}{q}}(\lambda_{1}) \le K$. Hence, $K = K_{1}^{\frac{1}{p}}(\lambda_{1})K_{2}^{\frac{1}{q}}(\lambda_{1})$ is the best possible constant factor of (23). The constant factor in (24) is still the best possible. Otherwise, we would reach a contradiction by (26) that the constant factor in (23) is not the best possible. □

## Operator expressions

With regard to the assumptions of Theorem 2, in view of

$$\begin{aligned}& c_{n}: = \frac{\prod_{k = 1}^{j_{0}} \nu_{n}^{(k)}}{ \Vert V_{n} \Vert _{\beta}^{j_{0} - p\lambda_{2}}} \biggl[ \sum _{m} \frac{(\min\{ \Vert U_{m} \Vert _{\alpha}, \Vert V_{n} \Vert _{\beta} \} )^{\eta}}{ (\max\{ \Vert U_{m} \Vert _{\alpha}, \Vert V_{n} \Vert _{\beta} \} )^{\lambda+ \eta}} a_{m} \biggr]^{p - 1}, \quad n \in\mathbb{N}^{j_{0}}, \\& c = \{ c_{n}\}, \quad\quad \Vert c \Vert _{p,\Psi^{1 - p}} = J < K_{1}^{\frac{1}{p}}(\lambda_{1})K_{2}^{\frac{1}{q}}( \lambda_{1}) \Vert a \Vert _{p,\Phi} < \infty, \end{aligned}$$

we can set the following definition.

### Definition 2

Define a multidimensional Hilbert’s operator $T:l_{p,\Phi} \to l_{p,\Psi^{1 - p}}$ as follows: For any $a \in l_{p,\Phi}$, there exists a unique representation $Ta = c \in l_{p,\Psi^{1 - p}}$, satisfying

27 $$ Ta(n): = \sum_{m} \frac{(\min\{ \Vert U_{m} \Vert _{\alpha}, \Vert V_{n} \Vert _{\beta} \} )^{\eta}}{(\max\{ \Vert U_{m} \Vert _{\alpha}, \Vert V_{n} \Vert _{\beta} \} )^{\lambda+ \eta}} a_{m}\quad \bigl(n \in\mathbb{N}^{j_{0}} \bigr). $$

For $b \in l_{q,\Psi}$, we define the following formal inner product of *Ta* and *b* as follows:

28 $$ (Ta,b): = \sum_{n} \biggl[ \sum _{m} \frac{(\min\{ \Vert U_{m} \Vert _{\alpha}, \Vert V_{n} \Vert _{\beta} \} )^{\eta}}{ (\max\{ \Vert U_{m} \Vert _{\alpha}, \Vert V_{n} \Vert _{\beta} \} )^{\lambda+ \eta}} a_{m} \biggr]b_{n}. $$

Then by Theorems 1 and 2, we have the following equivalent inequalities:

29 $$\begin{aligned}& (Ta,b) < K_{1}^{\frac{1}{p}}(\lambda_{1})K_{2}^{\frac{1}{q}}( \lambda_{1}) \Vert a \Vert _{p,\Phi} \Vert b \Vert _{q,\Psi}, \end{aligned}$$

30 $$\begin{aligned}& \Vert Ta \Vert _{p,\Psi^{1 - p}} < K_{1}^{\frac{1}{p}}( \lambda_{1})K_{2}^{\frac{1}{q}}(\lambda_{1}) \Vert a \Vert _{p,\Phi}. \end{aligned}$$

It follows that *T* is bounded with

31 $$ \Vert T \Vert : = \sup_{a( \ne\theta) \in l_{p,\Phi}} \frac{ \Vert Ta \Vert _{p,\Psi^{1 - p}}}{ \Vert a \Vert _{p,\Phi}} \le K_{1}^{\frac{1}{p}}(\lambda_{1})K_{2}^{\frac{1}{q}}( \lambda_{1}). $$

Since the constant factor $K_{1}^{\frac{1}{p}}(\lambda_{1})K_{2}^{\frac{1}{q}}(\lambda _{1})$ in (30) is the best possible, we have

32 $$ \Vert T \Vert = K_{1}^{\frac{1}{p}}(\lambda_{1})K_{2}^{\frac{1}{q}}( \lambda_{1}) = \biggl[ \frac{\Gamma^{j_{0}} ( \frac{1}{\beta} )}{\beta ^{j_{0} - 1}\Gamma ( \frac{j_{0}}{\beta} )} \biggr]^{\frac{1}{p}} \biggl[ \frac{\Gamma^{i_{0}} ( \frac{1}{\alpha} )}{a^{i_{0} - 1}\Gamma ( \frac{i_{0}}{\alpha} )} \biggr]^{\frac{1}{q}}k(\lambda_{1}). $$

### Remark 1

(i) For $\mu_{i} = \nu_{j} = 1$ ($i,j \in\mathbb {N}$), (23) reduces to (4). Hence, (23) is an extension of (4).

(ii) For $\eta= 0$, $0 < \lambda_{1} \le i_{0}$, $0 < \lambda_{2} \le j_{0}$, (23) reduces to the following inequality:

33 $$ \begin{aligned}[b] &\sum_{n} \sum _{m} \frac{1}{(\max\{ \Vert U_{m} \Vert _{\alpha}, \Vert V_{n} \Vert _{\beta} \} )^{\lambda}} a_{m}b_{n} \\ &\quad < \biggl[ \frac{\Gamma^{j_{0}} ( \frac{1}{\beta} )}{\beta^{j_{0} - 1}\Gamma ( \frac{j_{0}}{\beta} )} \biggr]^{\frac{1}{p}} \biggl[ \frac {\Gamma^{i_{0}} ( \frac{1}{\alpha} )}{a^{i_{0} - 1}\Gamma ( \frac{i_{0}}{\alpha} )} \biggr]^{\frac{1}{q}} \frac{\lambda}{ \lambda_{1}\lambda_{2}} \Vert a \Vert _{p,\Phi} \Vert b \Vert _{q,\Psi}. \end{aligned} $$

In particular, for $i_{0} = j_{0} = \lambda= 1$, $\lambda_{1} = \frac{1}{q}$, $\lambda_{2} = \frac{1}{p}$, (33) reduces to (5). Hence, (33) is also an extension of (5); so is (23).

(iii) For $\eta= - \lambda$, $\lambda_{1},\lambda_{2} < 0$, (23) reduces to the following inequality:

34 $$ \begin{aligned}[b] &\sum_{n} \sum _{m} \frac{1}{(\min\{ \Vert U_{m} \Vert _{\alpha}, \Vert V_{n} \Vert _{\beta} \} )^{\lambda}} a_{m}b_{n} \\ &\quad < \biggl[ \frac{\Gamma^{j_{0}} ( \frac{1}{\beta} )}{\beta^{j_{0} - 1}\Gamma ( \frac{j_{0}}{\beta} )} \biggr]^{\frac{1}{p}} \biggl[ \frac {\Gamma^{i_{0}} ( \frac{1}{\alpha} )}{a^{i_{0} - 1}\Gamma ( \frac{i_{0}}{\alpha} )} \biggr]^{\frac{1}{q}} \frac{( - \lambda)}{\lambda_{1}\lambda_{2}} \Vert a \Vert _{p,\Phi} \Vert b \Vert _{q,\Psi}. \end{aligned} $$

(iv) For $\lambda= 0$, $\lambda_{2} = - \lambda_{1}$ ($- \eta< \lambda_{1} < \eta$), (23) reduces to the following inequality:

35 $$ \begin{aligned}[b] &\sum_{n} \sum _{m} \biggl( \frac{\min\{ \Vert U{}_{m} \Vert _{\alpha}, \Vert V_{n} \Vert _{\beta} \}}{\max\{ \Vert U_{m} \Vert _{\alpha}, \Vert V_{n} \Vert _{\beta} \}} \biggr)^{\eta } a_{m}b_{n} \\ &\quad < \biggl[ \frac{\Gamma^{j_{0}} ( \frac{1}{\beta} )}{\beta^{j_{0} - 1}\Gamma ( \frac{j_{0}}{\beta} )} \biggr]^{\frac{1}{p}} \biggl[ \frac {\Gamma^{i_{0}} ( \frac{1}{\alpha} )}{a^{i_{0} - 1}\Gamma ( \frac{i_{0}}{\alpha} )} \biggr]^{\frac{1}{q}} \frac{2\eta}{ \eta^{2} - \lambda_{1}^{2}} \Vert a \Vert _{p,\Phi} \Vert b \Vert _{q,\Psi}. \end{aligned} $$

The above particular inequalities are also with the best possible constant factors.
